# Temperature Dependence of Hourly, Daily, and Event-based Precipitation Extremes Over China

**DOI:** 10.1038/s41598-018-35405-4

**Published:** 2018-12-03

**Authors:** Xichao Gao, Qian Zhu, Zhiyong Yang, Jiahong Liu, Hao Wang, Weiwei Shao, Guoru Huang

**Affiliations:** 1 State Key Laboratory of Simulation and Regulation of Water Cycle in River Basin, China Institute of Water Resources and Hydropower Research, Beijing, 100038 China; 2 China Institute of Water Resources and Hydropower Research, Beijing, 100038 China; 3 School of Civil Engineering, Southeast University, Nanjing, 211189 China; 4 School of Civil Engineering and Transportation, South China University of Technology, Guangzhou, 510640 China

## Abstract

Theoretically, precipitation extremes will increase at a rate of 6–7% with temperature increasing, namely the Clausius-Clapeyron relationship. However, many gauge observations suggest a peak structure of the relationship between precipitation extremes and atmospheric temperature, deviating from the Clausius-Clapeyron relationship. In this study, a comprehensive investigation about the temperature dependence of precipitation extremes (hourly, daily, and event-based) across China is implemented. The results confirm the widespread existence of the peak structure for daily and hourly precipitation extremes and show that (1) there is a generally positive spatial correlation between the precipitation extremes at the peak and temperature at the peak, and this scaling rate is close to the C-C rate; (2) the scaling of event-based extremes for precipitation amount with temperature follows a similar pattern to the daily precipitation extremes while the event-based precipitation intensity does not show a peak structure; (3) the decrease of rain duration is the main cause for the peak structure of the rain amount scaling.

## Introduction

Precipitation becomes more unevenly distributed in the warming climate forced by nature and anthropogenic activities. Historical observations and future predictions from global and regional climate models indicate that both the magnitude and the frequency of extreme precipitation increase obviously^1–5^, while the annual mean precipitation is detected to increase less than extreme precipitation or even decrease^6^, which may cause drought and flood risks^7,8^. Hence, prediction of precipitation, especially for precipitation extremes, is of significant importance to enhance the adaptivity of human beings to climate change. Among all the climate factors, temperature is the one better predicted than the others because of its physical characteristics and more accurate historical records^9^. Considering the predictability of temperature, predicting precipitation extremes based on the relationship between precipitation extremes and temperature is a feasible way under climate change. Therefore, detecting the fundamental processes of precipitation extremes with temperature is studied all over the world^10–12^.

Some studies point out that the temperature dependence of precipitation extremes is controlled by the Clausius-Clapeyron (C-C) relationship^9,13^. The explanation is that, theoretically, the distribution of relative humidity would remain roughly constant under climate change because of the control on the moisture in the troposphere: moisture condenses out of supersaturated air. In this case, the Clausius-Clapeyron (C-C) relationship implies that specific humidity would increase roughly exponentially with temperature at an approximate rate of 6–7% per degree Celsius. Therefore, under the assumption that the precipitation extremes tend to precipitate out all moisture in the rising air^9,13^, the relationship between precipitation extremes and atmospheric temperature (Ta) should follow the C-C relationship. However, many studies show that the relationship between precipitation extremes and Ta may not be always consistent with the C-C relationship. Lenderink and Meijaard^14^ find that the scaling rate is related to temperature in the Netherlands. The scaling rate of the temperature dependence of daily precipitation extremes is similar to the C-C rate at Ta below 8–10 °C and smaller than the C-C rate at higher temperatures, while that of hourly precipitation extremes is larger than the C-C rate (super-CC rate) at high temperatures. In Europe, a seasonality is detected in the temperature dependence of precipitation intensity with a general increase with temperature in winter and a decrease in summer^15^. Besides, peak structures, resulting from negative proportion in the relationship between precipitation extremes and Ta at higher temperatures, have been detected in many studies all over the world, especially for those areas located in mid-latitudes^12,16^. Utsumi *et al*.^16^ illustrate that the temperature dependence of daily precipitation extremes exhibited a peak-like structure at mid-latitudes (20°–55°N and S), and they point out that the decrease in the daily extreme precipitation intensity resulted from the decrease in the duration of the precipitation events. In Australia, Hardwick and Jones^17^ find that there would be an inflection in the relationship between precipitation extremes and surface temperature when the temperature is between 20 °C and 26 °C. They attribute the decrease in precipitation intensity at high temperatures (larger than 20–26 °C) to the decrease in relative humidity. In Canada, based on different duration observations and event-based analysis, Panthou *et al*.^18^ find that the duration and the climatic region are important factors that determine the relationship between precipitation extremes and temperature. They find a supper-CC scaling with an upper limit for those short duration observations in inland regions and illustrate that the relative humidity is a limitation for the relationship in inland regions but not for that in coastal regions. In China, the peak-like structures and supper-CC scaling are also found by Miao *et al*.^19^. In their work, peak structures of the relationship between hourly precipitation extremes and daily Ta are universal for all the first-class basins in China. The fact that the peak structures exist robustly all over the world probably indicate that there would be an upper limit for future precipitation extremes with the warming climate. However, Wang. *et al*.^13^. find that, compared with the historical temperature dependence of daily precipitation extremes, both the peak of extreme precipitation and the peak-point temperature will increase with warming climate, which means that this peak structure does not imply a potential upper limit for future precipitation extremes. As stated above, the relationship between precipitation extremes and Ta have been studied thoroughly, however, the relationship between peak-point extreme precipitation and peak-point temperature, the relationship between event-based precipitation extremes and temperature, and the relationship between precipitation durations and temperature are rarely investigated. The objectives of this paper were to (1) identify the relationship between peak-point precipitation extremes and peak-point temperature; (2) identify the relationship between event-based precipitation extremes and temperature; and (3) identify the relationship between precipitation durations of event-based precipitation extremes and temperature.

## Results

### Peak structure of temperature dependence of precipitation extremes

**Figure 1:**
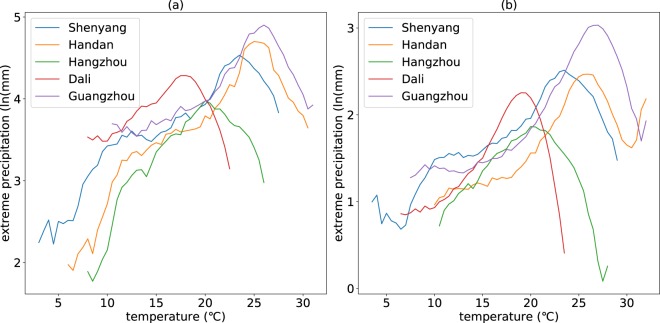
Temperature dependence of daily (**a**) and hourly (**b**) precipitation extremes for the randomly selected 5 stations. The figure shows the relationship between daily precipitation extremes and temperature (**a**) and the relationship between hourly precipitation extremes and temperature (**b**). The different colours represent different stations.

The daily and hourly precipitation extremes-Ta relationship are analysed over China. The results show that the temperature dependence of daily and hourly precipitation extremes for most stations (99.7% for both two temporal scales) have peak structures. We select 5 stations randomly, one for each climatic zone (severe cold region, cold region, hot summer and cold winter region, hot summer and warm winter region, and temperate region) over China, to illustrate the relationship curve patterns and the peak structures. Figure 1 shows that peak structure exists for all the randomly selected stations although the patterns of the relationship curves vary a lot. As to the scaling rates, only 13.6 percent stations have scaling rates close to C-C scaling rate (between 6.0% and 7.0%) for the temperature dependence of daily precipitation extremes and 27.8 percent stations for hourly precipitation. Most of the scaling rates (77.3% for daily temporal scale and 60.2% for hourly temporal scale) are larger than the C-C scaling rate which is similar to the study of Miao^19^. There is an abnormal phenomenon that more supper C-C relationship is found in daily precipitation extremes than hourly precipitation extremes, while, in many other studies, the supper C-C relationship is usually found in hourly precipitation extremes more than daily precipitation extremes. This phenomenon may be caused by the different regional climate characteristic (Europe or Asia), the different meteorological data sets (station-based or grid-based) and/or the different spatial scale (a point or a region).

#### Temperature dependence of peak-point precipitation extremes

**Figure 2:**
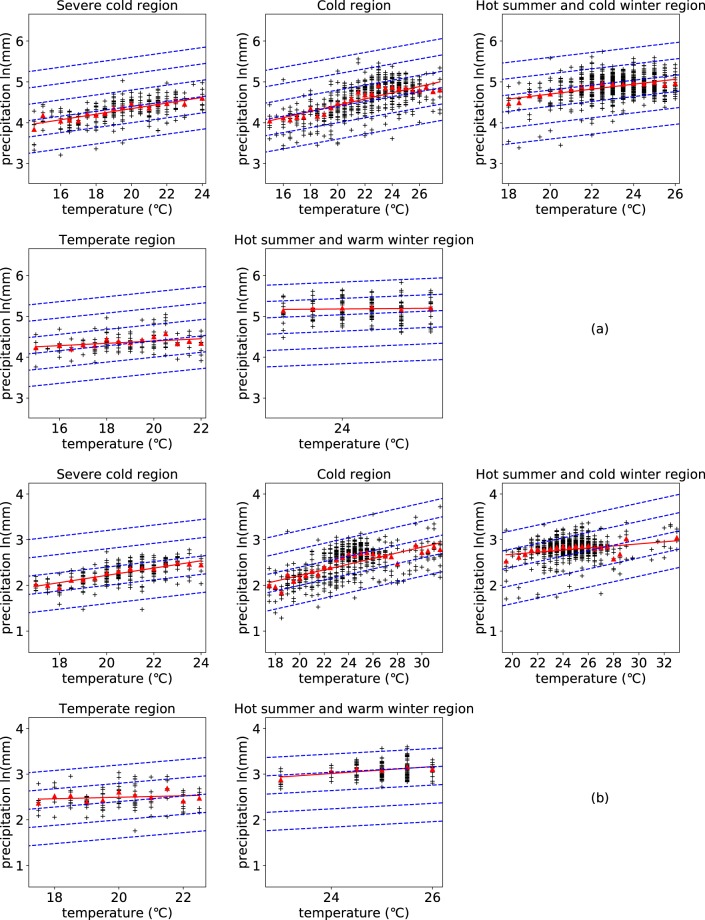
Relationship between peak points of daily temporal scale (**a**) and hourly temporal scale (**b**) in different climatic zones. The figure shows the relationship between peak-point precipitation extremes and peak-point temperature in different climatic zones at two temporal scales (daily (**a**) and hourly (**b**)). The red triangles represent the average peak-point extreme precipitation of corresponding temperature bins; The red line is the regression line of the relationship between the average peak-point extreme precipitation and the corresponding temperature; The dashed blue line represents the C-C relationship (6%/°C).

**Figure 3:**
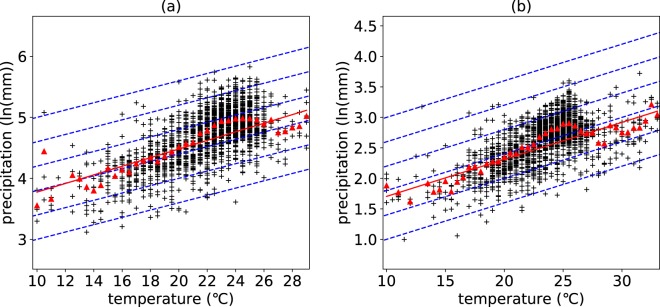
Relationship between peak points of daily temporal scale (**a**) and hourly temporal scale (**b**) across China. The figure shows the relationship between peak-point precipitation extremes and peak-point temperature at two temporal scales (daily (**a**) and hourly (**b**)). The red triangles represent the average peak-point extreme precipitation of corresponding temperature bins; The red line is the regression line of the relationship between the average peak-point extreme precipitation and the corresponding temperature; The dashed blue line represents the C-C relationship (6%/°C)).

To detect the relationship between different peak points of different stations, the peak-point precipitation and the corresponding peak-point temperature of all the stations are extracted. It should be noted that those temperature bins having less than 3 extreme precipitation points are ignored to eliminate the effect of anomalous values on the accuracy of the statistic results. The relationship between peak points is illustrated at different spatial scales: climatic zones and China. Figure 2 shows that the increasing rates of peak-point precipitation with corresponding increasing temperature vary a lot across different climatic zones and temporal scales. The increasing rates of daily peak points range from 0.012 to 0.076 while that of hourly peak points range from 0.016 to 0.080 (Table 1). It can be observed from Table 1 that the increasing rates of peak-point precipitation extremes with corresponding increasing temperature are close in severe cold region, cold region, and temperate region while deviate a lot in hot summer and cold winter region and hot summer and warm winter region. The large deviations may be due to the relatively small data samples of daily precipitation extremes compared with hourly data. From a perspective of China, most of the peak-point temperatures are distributed from 10 to 30 degrees, and the scaling rate of the relationship between daily (hourly) average peak-point extreme precipitation and the corresponding temperature is 7.0% (6.0%), consistent with the C-C scaling rate (Fig. 3). It should be noted that errors and uncertainties may be introduced by lumping the data together across different stations. Regional circulation pattern especially related to moisture supply has a significant influence on precipitation thermodynamics and dynamics. Lumping data across different stations controlled by different circulation patterns mixes different precipitation patterns and may lead to the scaling deviating from the C-C relationship.

**Table 1:** Scaling rates of peak points in different climatic regions.

Temporal scale	Severe cold region	Cold region	Hot summer and cold winter region	Temperate region	Hot summer and warm winter region
Daily	0.068	0.076	0.060	0.028	0.012
Hourly	0.080	0.062	0.022	0.016	0.076

The table gives the values of the scaling rates between peak-point precipitation extremes and peak-point temperature in different climatic regions.

**Figure 4:**
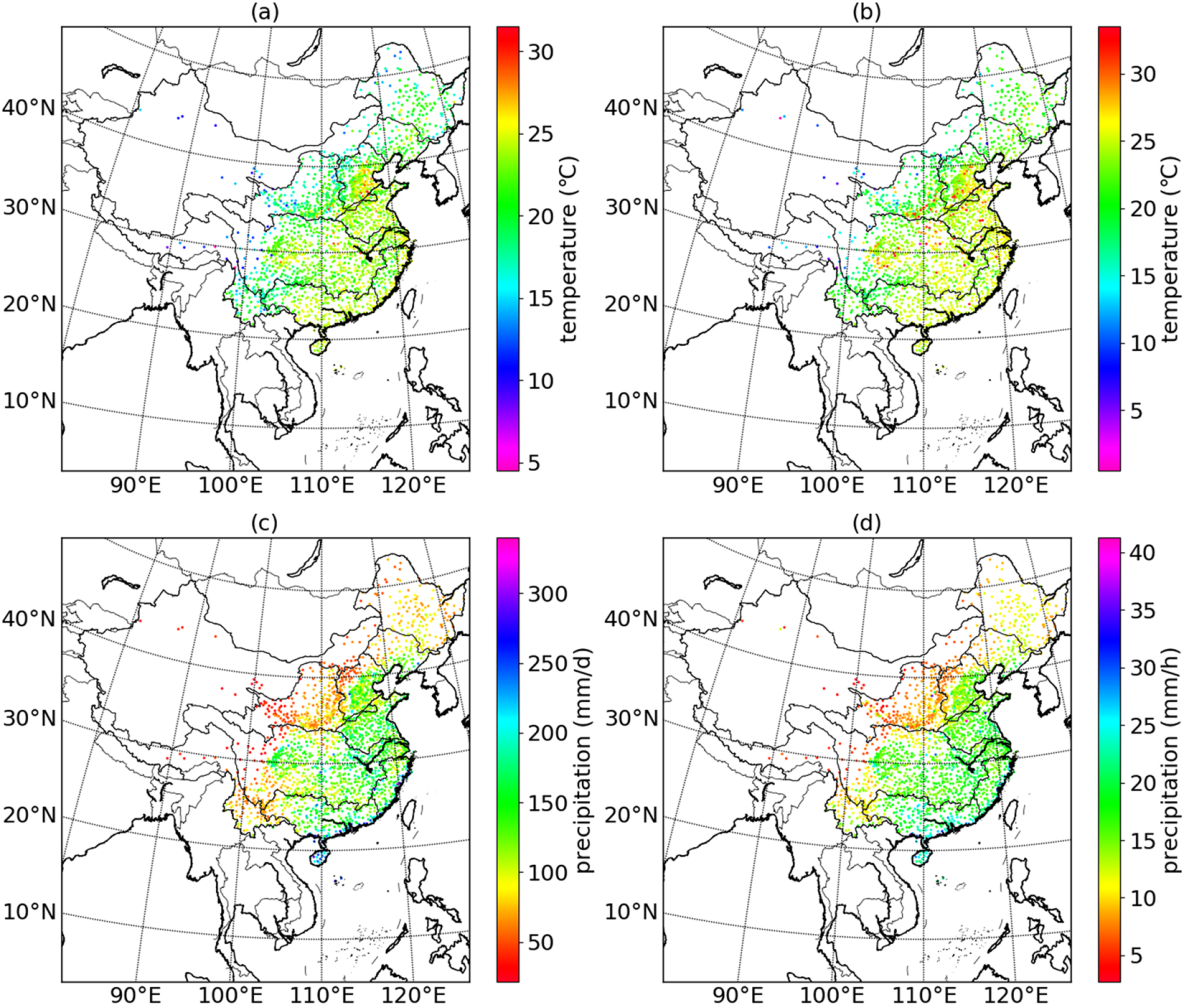
Spatial distribution of peak-point temperatures and peak-point extreme precipitation of daily temporal scale and hourly temporal scale. (**a**–**d**) Represents the spatial distribution of daily peak-point temperatures, hourly peak-point temperatures, daily peak-point precipitation, and hourly peak-point precipitation, respectively.

The spatial distributions of peak-point temperatures and the peak-point precipitation extremes of daily temporal scale and hourly temporal scale are shown in Fig. 4. The peak-point temperatures of both temporal scales show an upward trend roughly along the normal direction of the “Hu line”^20^. The “Hu line”, starting from Aihui County in the northeast of China to Tengchong City in the southwest of China, divides China into densely-populated rich southeast region and underpopulated poor northwest region. The line is highly consistent with the 400 mm precipitation isoline of China. The spatial distribution of the peak-point precipitation extremes is similar to the distribution of the peak-point temperatures, increasing from northwest to southeast along the normal direction of the “Hu line”. The spatial distribution patterns of the peak points verifies the trend shown in Fig. 1 that the peak-point extreme precipitation increases with the increasing peak-point temperature.

#### Relationship between event-based precipitation extremes and Ta

**Figure 5:**
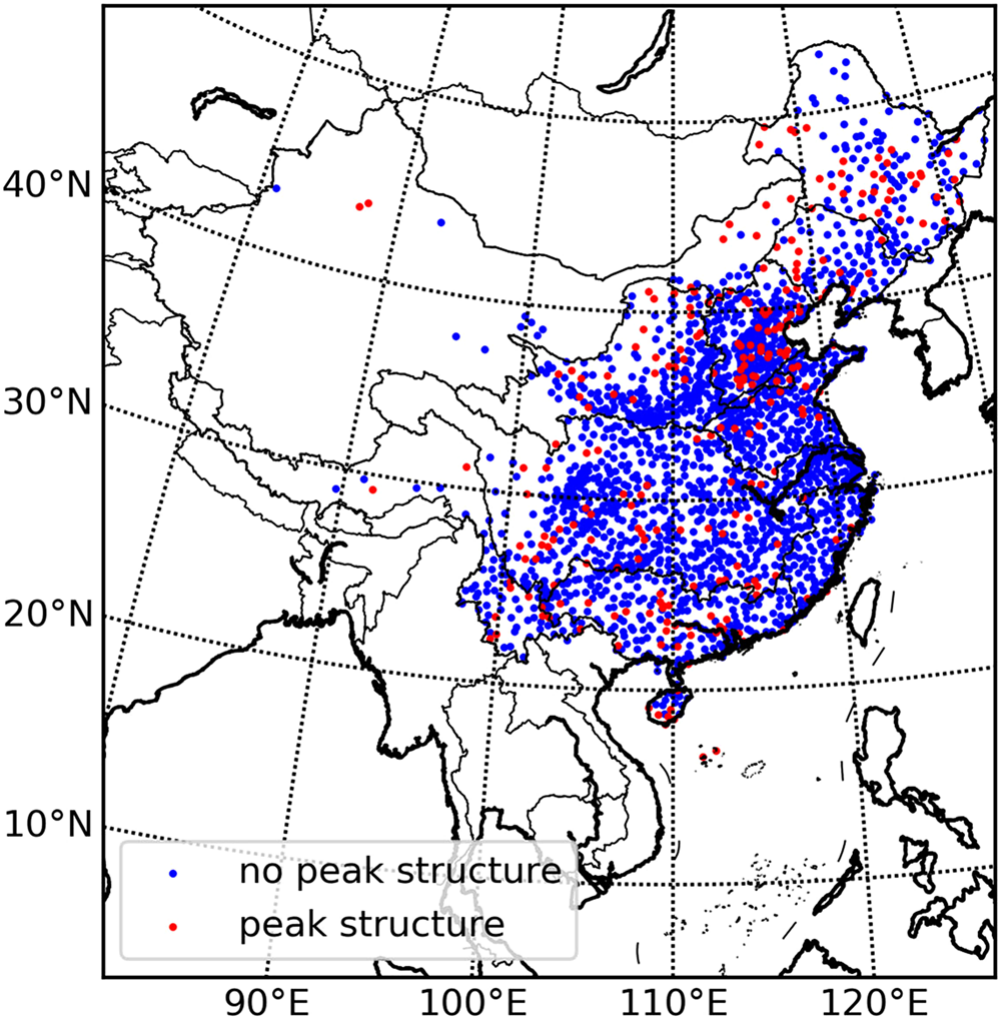
Spatial distribution of stations with/without peak structure in the relationship between PIE and Ta. The figure shows the relative spatial distribution of stations with or without peak structure in the relationship between PIE and Ta. The red points represent stations with peak structure while the blue points represent stations without peak structure.

**Figure 6:**
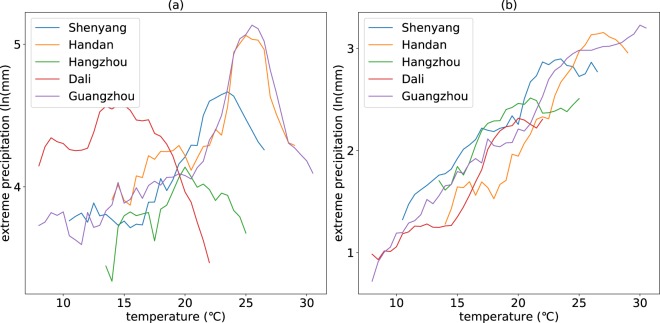
Temperature dependence of PAE (**a**) and PIE (**b**) for the randomly selected 5 stations. The figure shows the relationship between PAE extremes and temperature (**a**) and the relationship between PIE and temperature (**b**). The different colours represent different stations.

To explore the generation mechanism of the peak structure of temperature dependence of precipitation extremes, event-based analysis is performed from two perspectives: the relationship between precipitation event amount extremes (PAE) and Ta, and the relationship between precipitation event mean intensity extremes (PIE) and Ta. The temperature dependence of PAE and PIE takes on different trends. The relationship between PAE and Ta have peak structures in almost all the stations, while the relationship between PIE and Ta shows a monotonous increase trend in the overwhelming majority of the stations (88.2%). The spatial distribution of stations that have no peak structures and have peak structures is illustrated in Fig. 5. The stations where peak structures are found in the relationship between PIE and Ta are mainly located in severe cold region. The detailed statistic information for stations having or not having peak structures is shown in Table 2. More than 80% stations do not have peak structures in the relationship between PIE and Ta for most climatic zones except severe cold region (76.1%), which demonstrates that the characteristic of monotonous increase in the temperature dependence of the PIE is relatively robust for most climatic zones all over China. The scaling rates are not consistent with the C-C relationship in most stations. Only 23.2 percent stations have scaling rates close to C-C scaling rate (between 6.0% and 7.0%) for temperature dependence of PAE and 11.3 percent stations for PIE. Similar to the scaling rates of observations, most of the scaling rates for the event-based precipitation extremes are larger than the C-C scaling rate (56.5% for the PAE and 83.1% for the PIE). Figure 6 shows the temperature dependence of PAE and PIE for the selected 5 stations mentioned above. The relationships between the PAE and Ta show an obvious peak structure while the relationship between the PIE and Ta do not have peak structures.

**Table 2:** Spatial statistics of stations with/without Peak Structure in temperature dependence of PIE.

Climatic zones	Stations with peak structure	Stations without peak structure	The proportion of no-peak stations (%)
Severe cold region	68	216	76.1
Cold region	73	551	88.3
Hot summer cold winter region	48	772	94.1
Temperate region	18	113	86.3
Hot summer warm winter region	34	149	81.4
Total	241	1801	88.2

The table gives the count of stations with and without peak structure in the relationship between PIE and temperature respectively for different climatic regions.

**Figure 7:**
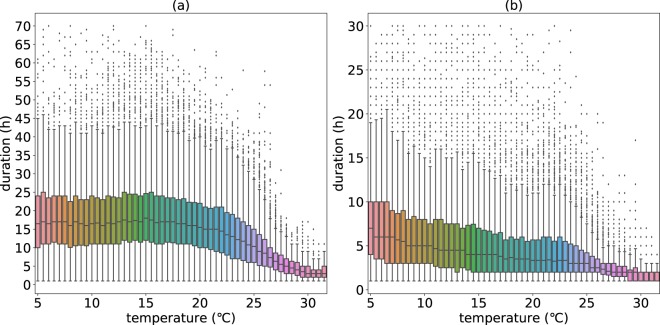
Temperature dependence of durations of PAE (**a**) and PIE (**b**). The figure shows the relationship between PAE durations and temperature (**a**) and the relationship between PIE durations and temperature (**b**). The boxes indicate the 25th, 50th, and 75th percentiles of the distribution, and the vertical lines indicate the 5th and 95th percentiles.

Rainfall duration is a critical factor in distinguishing rainfall types. Generally, large-scale rainfall caused by the slow ascent of air in the synoptic system has long precipitation duration while convective rainfall falls over a certain area for a relatively short time^21^. To figure out the mechanism of the peak structures, the temperature dependence of the duration of PAE and PIE is investigated. As Fig. 7(a) described, the durations of PAE decrease dramatically from daily scale to hourly scale at high temperatures (around 25 °C), which imply that the rainfall type changes from the one dominated by a large-scale type to the one dominated by a more convective type. However the durations of PIE are mainly within sub daily scale and show less fluctuation, implying an consistently convective rainfall type (Fig. 7(b)).

**Figure 8:**
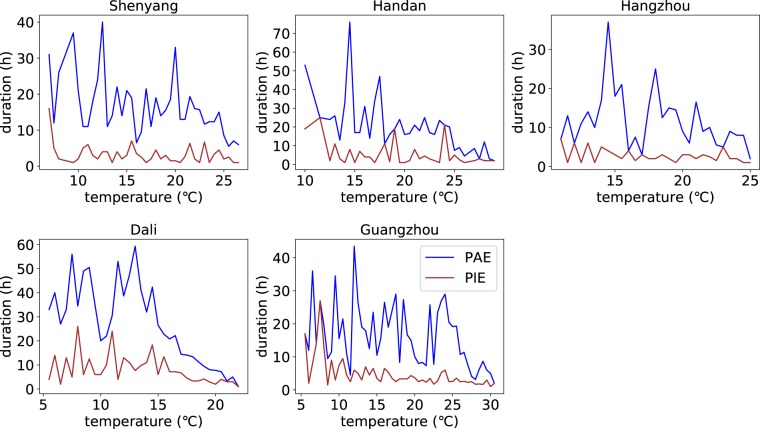
Temperature dependence of durations of PAE and PIE for the randomly selected 5 stations. The figure shows the comparison of the changes of PAE durations and PIE durations with temperature increasing. The blue lines represent the durations of PAE and the red lines represent the durations of PIE.

To illustrate the difference between PAE and PIE more obviously, The durations of those randomly selected 5 stations above are compared. Figure 8 illustrates that the duration of PAE and PIE show different patterns, which means that, in most cases, the PAE and the PIE are not the same processes at same temperatures. Generally the durations of PAE are longer than PIE and show a sharp decrease around high temperatures, which implies that the rainfall type changes from the one dominated by large-scale rainfall to the one dominated by more convective rainfall, while the duration of PIE shows little dependence on temperature.

## Discussion

**Figure 9:**
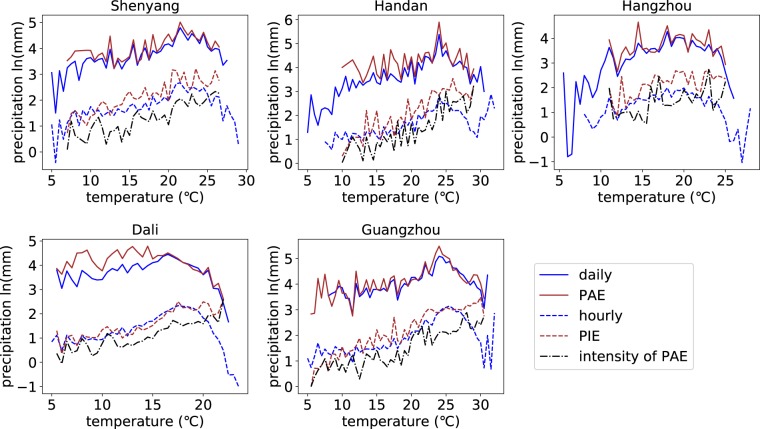
Comparison of daily (hourly) precipitation extremes and PAE (PIE) for the randomly selected 5 stations. The figure shows the comparison between the daily precipitation extremes and PAE as well as the comparison of hourly precipitation extremes, PIE, and intensity of PAE.

Noted that the magnitude of daily precipitation extremes and PAE are almost the same (Fig. 9) and the duration of PAE ranges from daily scale to hourly scale (Fig. 8), the characteristics of PAE, to some extent, can help to detect some dynamic characteristics of daily precipitation extremes. From Figs 8 and 9, it can be seen that the decreasing pattern in daily precipitation extremes is similar to that in the duration of PAE. For example, at station Guangzhou, daily precipitation extremes show a decreasing trend at high temperature around 23–25 °C, which is consistent with the steep decrease in the duration of PAE of which the shift point is also located in 23–25 °C. Besides, the average intensity of PAE increases with temperature increasing or levels off at high temperatures (Fig. 9). Hence, we attribute the peak structures in the relationship between daily precipitation extremes and Ta to the decrease in rainfall duration, which is due to the change of rainfall type from large-scale precipitation to convective precipitation at high temperatures. Some other studies^17,18,22^ find that the relative humidity decrease at high temperatures in many regions all over the world and argue that the available moisture is the control factor for the decrease of daily precipitation extremes at high temperatures. However, the mechanism of the interaction with the atmospheric saturation level is not clear - the atmospheric saturation level can be both a cause and a consequence of the extreme precipitation^16^.

The temperature dependence of PIE shows different trend compared with that of hourly precipitation extremes (Fig. 5). PIE shows an increasing trend with temperature going up, while the hourly precipitation extremes display an increasing trend followed by a sharp decrease at high temperatures. It is an unexpected phenomenon as the magnitude and the duration of hourly precipitation extremes and PIE are similar. The reasons for this phenomenon could be as follows: (1) the sample of event-based precipitation extremes is much smaller than that of hourly observation extremes at very high temperatures so that the sample size is not enough to acquire the 99 quantiles, and (2) since the most extreme precipitation often occur at midnight^19^ and we take the mid-time temperature as the lateral axis for PIE, the temperature axis of hourly observation extremes and PIE may be different considering the cooling effect of the heaviest precipitation. Taking a 5-hour rainfall as an example, if 2-hour rainfall with much bigger intensity occurs before 24 o’ clock and 3-hour rainfall that has slight intensity occurs in the next day, the temperature of the next day will be used as the lateral axis of PIE and the temperature of the previous day will be used as the lateral axis of hourly precipitation extremes. In this case, the temperature axis of PIE may be not reasonable for the cooling effect of the heaviest precipitation is ignored. In other words, the temperature of PIE used in the statistic method may be higher than the actual one. Some recent studies^23^ indicate that, to some extent, high-temperature weathers are accompanied by various types of circulation anomaly such as anomalous anticyclone in China. Anticyclonic conditions bring dry weather, reduce relative humidity, and inhibit deep convection^24^, leading to the decrease of hourly precipitation extremes with warming at very high temperatures. According to the analysis above, the downturn in the scaling of hourly precipitation extremes in China may be caused by following reasons: (1) the cooling effect of the heaviest rainfall, (2) the much more anticyclone bringing hotter and drier weathers.

## Data and Methods

### Data

Hourly precipitation dataset is used in this study, which is provided by the National Meteorological Information Centre of the China Meteorological Administration. Daily mean temperature data is obtained from the SURF_CLI_CHN_MUL_DAY_V3.0 dataset, downloaded from the China Meteorological Data Sharing Service System (http://cdc.nmic.cn/home.do). 20 National stations are used for providing these precipitation and temperature datasets, which is selected from more than 2400 stations in the dataset considering the length of the sequences (longer than 30 years).

### Methods

Theoretically, the extreme precipitation increases with temperature following the Clausius-Clapeyron relationship (on a rate of 6–7%/°C)^9^. The scaling rate can be estimated as^17^:

1 $${P}_{b}={P}_{a}{\mathrm{(1}+r)}^{({T}_{b}-{T}_{a})}$$

In which, *P*_*a*_ and *P*_*b*_ are precipitation of time a and time b while *T*_*a*_ and *T*_*b*_ are atmospheric temperature of time a and time b, respectively. *r* is the scaling rate, of which theory value is 6–7% per Celsius degree^25^.

The temperature dependence of daily and hourly precipitation extremes is analysed to identify the peak structure in the relationship between precipitation extremes and Ta suggested by many studies^26^. The peak-point temperature and the corresponding precipitation extreme are extracted using the locally weighted regression smoothing (LOWESS) method^27^. To explore the temperature dependence of precipitation extremes in details, the event-based analysis is performed from two perspectives: one is the relationship between precipitation event amount extremes (PAE) and Ta, and the other is the relationship between precipitation event mean intensity extremes (PIE) and Ta. Precipitation events are usually derived from precipitation observations by specifying a minimum precipitation event amount and a minimum inter event time^28^. However, Panthou *et al*.^18^ indicate that the definition of these two parameters has no significant impacts on the results through a sensitivity analysis of the two parameters. Hence, in this study, we define the precipitation event as the continuous hours when precipitation depth is larger than 0.3 mm simply.

The temperature is first divided into bins with the size of 0.5 °C. Then precipitation is allocated to bins according to the temperature when it occurs. Within each Ta bin containing more than 50 data points, the 99th percentile of precipitation is estimated. For each temperature bin, the precipitation exceeding the 99th percentile is averaged and paired with the temperature of the middle of the bin. Those bins with data point less than 50 are discarded. The resulting extremes corresponding to different bins are then smoothed with 3-bin moving window averaging^13^. Then, the resulting statistics are used to explore the relationship between precipitation extremes and Ta.
